# Diabetic Foot Assessment using Skin Impedance in a Custom Made Sensor-sock

**DOI:** 10.2478/joeb-2022-0019

**Published:** 2023-01-14

**Authors:** Christian Tronstad, Maryam Amini, Eline Olesen, Elisabeth Qvigstad, Oliver Pabst, Tormod Martinsen, Sisay M. Abie, Ørjan G. Martinsen, Jonny Hisdal, Trond G. Jenssen, Håvard Kalvøy

**Affiliations:** 1 Department of Clinical and Biomedical Engineering, Oslo University Hospital, Oslo, Norway; 2 Department of Physics, University of Oslo, Oslo, Norway; 3 Department of Endocrinology, Morbid Obesity and Preventive Medicine, Oslo University Hospital, Oslo, Norway; 4 Institute of Clinical Medicine, University of Oslo, Oslo, Norway; 5 Faculty of Ecology and Natural Resource Management, Norwegian University of Life Sciences, Oslo Ås, Norway; 6 Department of Vascular Surgery, Oslo University Hospital, Oslo, Norway; 7 Department of Transplantation Medicine, Oslo University Hospital, Oslo, Norway

**Keywords:** Skin impedance, diabetes, diabetic foot, neuropathy

## Abstract

Diabetic peripheral neuropathy (DPN) may lead to several changes in the skin, and some of these may influence the skin impedance spectrum. In the present study we have developed a prototype solution for skin impedance spectroscopy at selected skin sites (big toe pulp, heel and toe ball) that was tested in a pilot study on five patients with DPN and five healthy controls. At the big toe, most of the controls had markedly lower impedance than the DPN group, especially in the range of 1-100 kHz. The separation between the groups seems to be weaker at the heel and weakest at the toeball. The results may indicate that monitoring of the skin impedance spectrum may be a method for detection of skin changes associated with DPN, encouraging further studies with the big toe sensor in particular.

## Introduction

Diabetic foot syndrome is an important cutaneous manifestation occurring in 15-25% of patients with diabetes, in which diabetic peripheral neuropathy (DPN) and angiopathy play crucial roles in its development [Behm et al. 2012]. Diabetic peripheral neuropathy (DPN) is the most common acquired neuropathy and one of the main complications of diabetes due to high prevalence, hospitalizations, morbidity and mortality [Fateh et al. 2016]. An early and accurate diagnosis is essential to reduce risk of complications [Yang et al. 2020, Yorek et al. 2018], but can be challenging given that almost half of the cases with DPN are symptom-free [Pop-Busui al 2016]. A wide variety of screening tools are available for DPN [Brown et al. 2017, Bönhof et al. 2019], that test different properties related to nerve function such as nerve conduction, sensation to vibration and sudomotor function. There are also changes occurring in the skin that for some may precede the diagnosis of diabetes [Brostow et al. 2008]. In at least one third of all persons with diabetes, long-standing diabetes may impair skin homeostasis resulting in skin manifestations [Behm et al. 2012].

Bioimpedance is a non-invasive method for measuring the passive electrical properties of tissue and has been studied for several applications in dermatology such as skin cancer identification [Åberg et al. 2004], skin hydration [Martinsen et al. 2008, Morin et al. 2020] and transdermal drug delivery [Arpaia et al. 2017, Clemente et al. 2013]. With respect to the diabetic foot and DPN there are several known skin changes that in theory may influence the skin impedance such as: dry skin, thinning of the epidermis, cracked skin and development of calluses. Dryness of skin is common in the diabetic foot, where autonomic neuropathy may be a significant cause for anhidrosis, predisposing individuals to fissuring and cracking of the epidermis, which in turn can lead to secondary infections [Bristow et al. 2008]. The thickness of the epidermal layer of plantar skin has been found to be decreased in people with diabetic foot ulceration and neuropathy [Chao et al. 2011]. Abnormal pressure on the foot may increase keratinization of skin cells developing into a callus, which predisposes to foot ulceration [Arosi et al. 2016].

Previous studies have indicated potential to detect skin differences between people with diabetes and healthy controls using bioimpedance spectroscopy. In Lindholm-Sethson et al. 1998, skin impedance spectra from four different skin sites (dorsal foot, leg, hand and arm) were measured in 16 participants with Type 1 Diabetes and 12 healthy controls. Mutivariate analysis of the impedance spectra revealed promising discrimination between the groups. Later on, the same research group combined skin impedance spectra with near-infrared spectroscopy in a larger study, obtaining an 85% classification accuracy [Nystrøm et al. 2003]. Prado-Olivarez et al. (2015) measured skin impedance spectra from the hallux of participants with and without diabetes and found a distinctive behavior in the phase angle at the frequency range of 1 kHz-20 kHz. Torres et al. 2018 presented an instrument to measure foot sole skin impedance and temperature, indicating from preliminary results significant impedance differences in the range of 5-22 kHz.

In this paper, we present an early prototype for skin impedance measurement in a custom made sensor-sock, and data from the first tests in a pilot study on participants with DPN without ulcers and healthy controls. The main aim of the study was to compare skin impedance spectra between the two groups, in addition to compare the suitability of different skin sites.

## Materials and methods

### Skin impedance electrode

**Figure 1:**
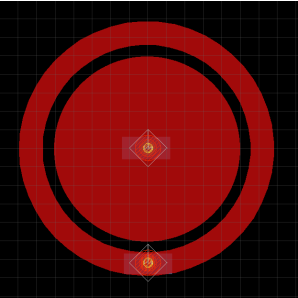
Screenshot from Eagle CAD design showing the electrode geometry (each grid unit is 1mm).

A previous study compared different variants of three types of electrode geometries (concentric ring electrodes, row electrodes, interdigitated array electrodes and unipolar electrodes) with respect to sensitivity and repeatability of plantar skin impedance measurement, and found a bipolar ring electrode (as shown in figure 1) to be the best suited candidate for this purpose [Olesen 2020]. The small gap between the inner and outer electrodes (0.5mm) maintains focus of the impedance measurement to the skin (mainly the epidermis) up to high frequencies, and the circular design may average out anisotropic effects thus possibly providing better repeatability of measurements.

**Figure 2:**
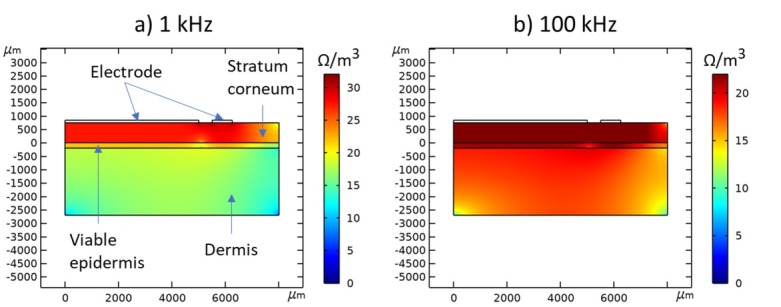
Axisymmetric 2D distribution of simulated volume impedance density below the electrode for low (a) and high (b) frequency impedance measurement. The color gradient indicates the volume impedance density, presented on a logarithmic scale.

In order to assess an approximate contribution from the different skin layers to the impedance measurement, we used Comsol Multiphysics® to calculate the volume impedance density below the electrode in a simulated measurement at a low (1 kHz) and high (100 kHz) excitation frequency. Values for the thickness of plantar stratum corneum and viable epidermis was acquired from [Boyle et al 2019] and values for the resistivities and permittivities of the skin layers were acquired from [Grimnes and Martinsen 2015, Tsai et al 2019]. The simulations shown in figure 2 indicate that the measurement is dominated by the epidermal stratum corneum at both low and higher frequencies.

The electrode was drawn in Eagle (Autodesk Inc, CA, USA) and fabricated by printing of silver ink (Voltera Inc, ON, Canada) on FR4 boards using a Voltera V-One PCB printer (Voltera Inc, Canada) followed by curing and polishing of the surface. Electrode leads were soldered to via connectors at the backside of the PCB and sealed by epoxy.

### Sensor sock prototype

**Figure 3:**
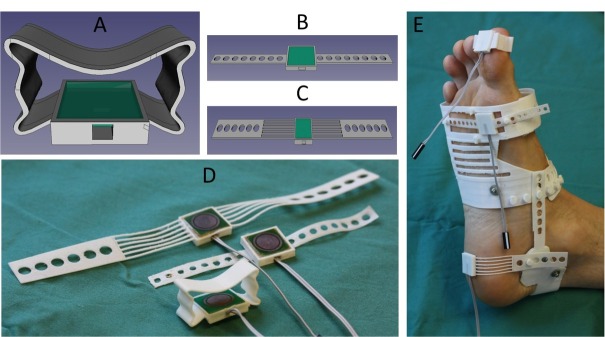
CAD design of sensor parts for skin impedance measurement at the big toe (A), toeball (B) and heel (C). The 3D printed parts for sensor housing are shown in D, and all parts connected to the foot are shown in E.

The housing parts for attachment to different skin sites (big toe, toe ball and heel) shown in figure 3 A-C were designed in FreeCAD and 3D printed with a soft and elastic plastic material (thermoplastic polyurethane) with a Shore hardness of 85A (Ninjaflex, Ninjatek, PA, USA). The geometry and softness of the materials were selected in order to provide a gentle pressure on the skin with minimal variation between different feet. While the toe part (electrode + housing) could be attached separately around the toe, the toe ball and heel parts were made to be connected to a soft plastic sock with straps (figure 2 D) for soft but stable contact to the skin. The sock was also designed in FreeCAD and 3D printed with a soft and elastic plastic material using thermoplastic polyurethane with a Shore hardness of 95A (Cheetah, Ninjatek. PA, USA). With this prototype, we could measure the skin impedance spectra at these selected skin sites with stable contact for different foot sizes and shapes.

### Pilot study

A pilot study was performed at the Diabetes Laboratory, Oslo University Hospital, Aker, Norway. Five patients, diagnosed with DPN (4♂/1♀) but without ulcers, and five healthy age-matched controls (2♂/3♀) were recruited for the study. The age distributions of the DPN group and the control group were 64.0±14.4 years and 60.6±11.1 years respectively.

Glycosylated haemoglobin (HbA1c), currently the main diagnostic test for diabetes, was initially measured in order to exclude potentially undiagnosed diabetes among the control participants. This was followed by the monofilament test [Dros et al. 2009] done by an experienced diabetes nurse at the laboratory. The right foot of the participant was then measured using selected non-invasive sensors including the presented skin impedance measurement. The skin impedance measurement was done immediately after electrode attachment – first at the toe pulp, then at the toe ball and finally at the heel. The skin was cleaned with an alcohol-based sanitizer just over an hour prior to the impedance measurement. During measurements, participants relaxed in a comfortable chair with legs resting horizontally on a foot rest, and were free to chat. This was part of a study using different types of non-invasive methods to assess DPN. Skin impedance frequency sweeps from 10 Hz to 5 MHz was done using a Zurich Instruments MFIA (Zurich Instruments AG, Switzerland) connected to the electrodes with a two-electrode configuration. Shielded cables with a length of 1.4 m were used between the MFIA device and the sock. For electrical safety, the impedance analyzer was run on battery power and an optocoupler was used in the USB connection to a battery-powered laptop controlling the measurement. The electrodes were cleaned with peracetic acid and ethanol between each participant.

To estimate the measurement repeatability and compare between skin sites, each site was measured three times successively with the electrode detached and reattached between the measurements. The impedance modulus at the lowest frequency was used to estimate a percentwise repeatability for each skin site as:



$$R_{\text{subject },\text{ site }}=100\cdot \frac{\mathrm{std}\left(\log_{10}\left(|Z{|}_{10\;Hz}\right)\right)}{\mathrm{mean}\left(\log_{10}\left(|Z{|}_{10\;Hz}\right)\right)}$$

### Informed consent

Informed consent has been obtained from all individuals included in this study.

### Ethical approval

The study was approved by the regional ethics committee (REK#152845), and the appointed committee at Oslo University Hospital evaluated and approved the measurement setup in accordance with Annex VIII, to be applicable with the European Medical Devices Directive— 93/42/EEC with 2007/47/EC.

## Results

**Figure 4:**
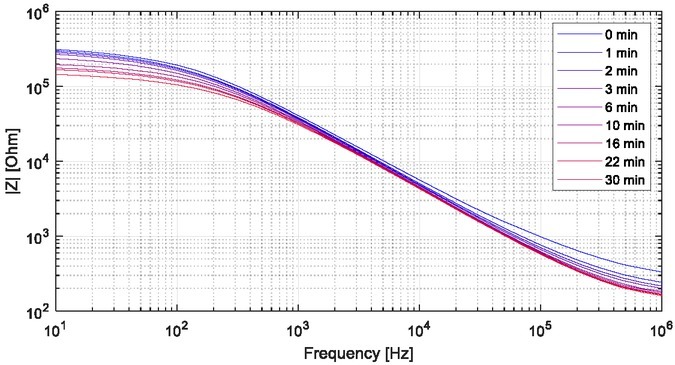
An example measurement of change in measured skin impedance over 30 minutes using the electrode probe at the big toe pulp of a healthy subject. These measurements were done with the Solartron 1260+1294.

Figure 4 shows a typical measurement with the toe electrode on a healthy subject, also showing how the impedance can change over time (30 minutes) after electrode contact.

**Figure 5:**
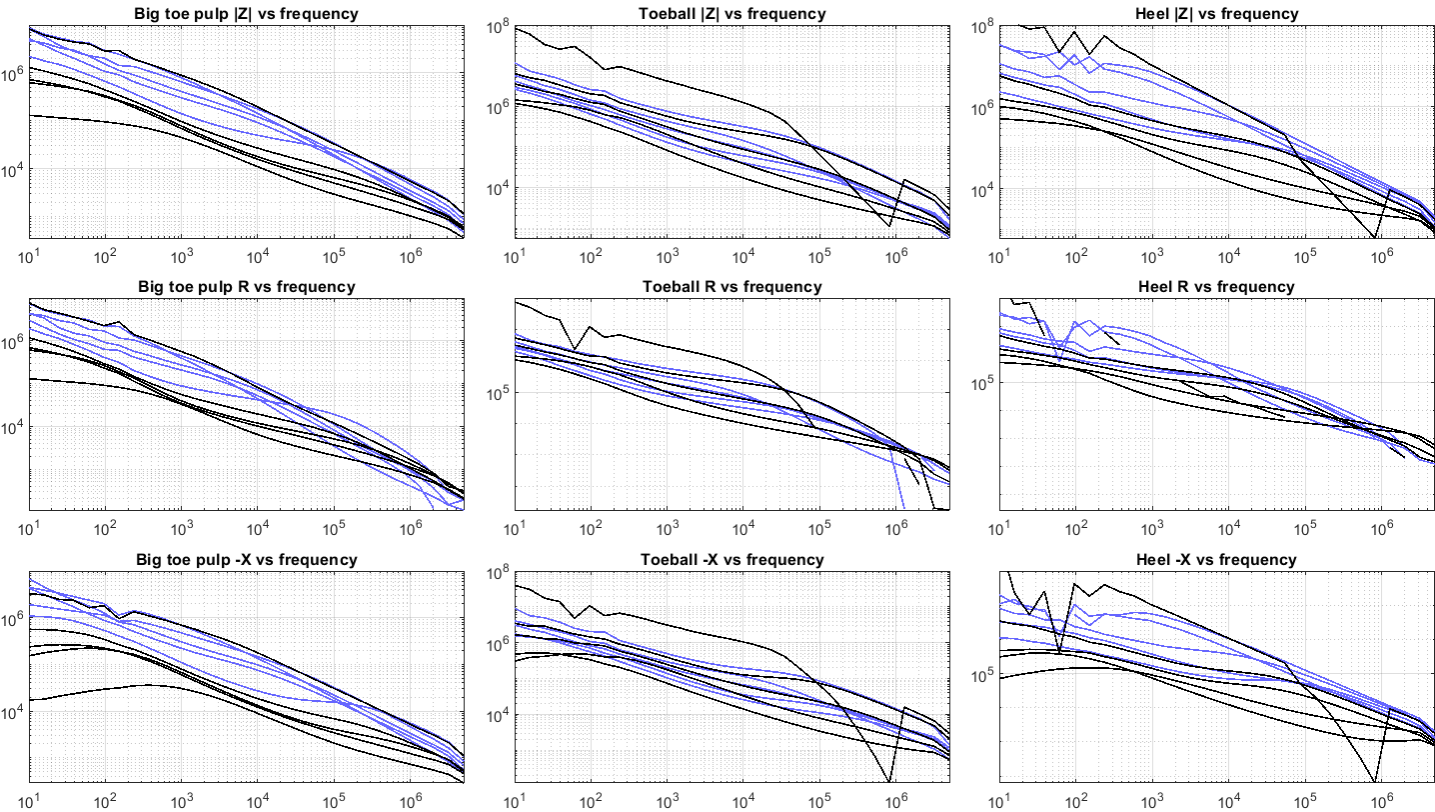
Impedance spectra presented as the impedance modulus (|Z|), resistance (R) and reactance (X) for all participants from the control (black) and DPN group (blue). The bumpy curves with the highest impedance in the toeball and heel plots curves are affected by measurement error due to the high impedance levels and do not represent a true frequency-dependency of the skin impedance.

Figure 5 summarizes the skin impedance spectra (the first out of the three repeated measurements) for all skin sites for both groups of participants, represented as the impedance modulus, resistance and reactance.

**Figure 6:**
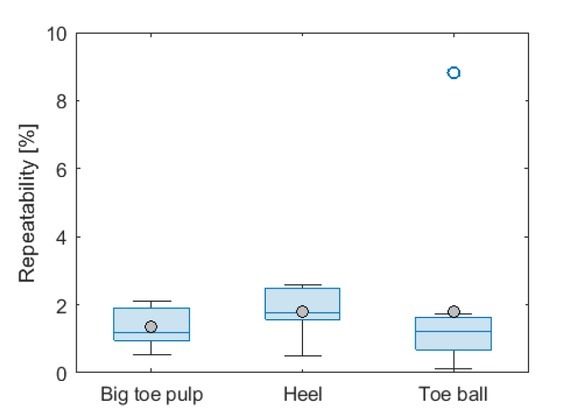
Comparison of the repeatability of all measurements (control and DPN groups combined) between the different skin sites presented in a boxplot with group means added in grey dots. White dots represent outliers and edges of the whiskers represent the minimum and maximum non-outlier values within the groups. Repeatability was calculated using the log10-transformed impedance modulus at 10 Hz, taking the standard deviation of the successive measurements divided by the mean.

## Discussion

In most of the cases, we found markedly differences between the DPN and control group in the skin impedance spectra from the big toe and the heel, while at the toeball the distribution in skin impedance had a large overlap between the groups. The strongest separation between the groups was seen for the mid-range frequencies (10^3^-10^5^ Hz) at the big toe skin site. Measurement repeatability was comparable between the skin sites, but slightly better for the big toe pulp. For the big toe pulp measurement, all but one control subject had a lower skin impedance over all frequencies compared to the DPN group. Due to the limitation of this pilot study, we cannot say for certain whether this one case was due to different skin properties or a technical cause such as electrode contact. In some cases, the skin impedance was reaching the GΩ range for the lowest frequencies, which was higher than expected from preliminary testing before the study. Based on the |Z| accuracy specification of the MFIA, the instrument accuracy is still within the 1 % range for this impedance range at low frequencies. The differences between the groups may be caused by increased dryness of the plantar skin in the DPN group, which is a known effect of DPN [Nikoleishvili et al. 2006].

For skin impedance measurement using dry electrodes, it is essential to maintain a soft but stable contact with the skin in order to reduce errors from pressure variations or poor electrical contact. After placement of the electrodes on the skin, the measured impedance changes over the first minutes, due to accumulation of perspiration between the electrodes and skin, improving the electrode contact and reducing the impedance [Geddes et al. 1973]. Figure 4 shows an example of this effect for the electrode used in this study. In this study, we considered a measurement directly after electrode attachment to provide the most inert skin impedance measurement (without the electrode effects) compared to measuring some time after electrode attachment or after the electrode effects have completely stabilized. We do not know whether a measurement before or after electrode stabilization would provide the best measurement with respect to diabetic foot skin assessment, but a quick test (not requiring much time for stabilization) is desired with a clinical application in mind. One measurement (10 Hz to 5 MHz impedance sweep) takes just a few seconds to complete, and electrode effects during the measurement after electrode attachment would be small.

We also gathered the following experiences from the pilot study:

- Impedance levels measured at plantar skin could be much higher than anticipated based on preliminary testing of the electrode.
- The geometry of the toe ball made it more difficult to position the electrode at a distinct place, compared to the big toe and heel.
- None of the participants reported the sock as uncomfortable

It would have been of interest to also include lower frequencies than 10 Hz in the measurement in order to provide more information and possibly cover the alpha dispersion in the frequency spectrum. However, lower frequencies demand relatively high measurement time during which electrode effects may occur. This study did not consider natural interindividual variations in the thickness of skin layers, which is a potential source of error. Furthermore, this pilot study is limited by the small sample size, no blinding, limited selection of skin sites and testing of only one particular type of electrode.

At this point based on the results from this small study, it is yet unclear whether skin impedance spectroscopy may be useful in DPN detection, DPN grading in the skin, or ulceration risk estimation compared to other simple tests. However, the results from the present study indicate a potential to detect relevant skin changes associated with DPN, and with methodological improvements, the toe sensor in particular can be included in further studies. The toe site may also be the most relevant site to detect early changes, as DPN is known to begin distally and progress proximally [Dobretsov et al. 2007, Iqbal et al. 2018]. In a larger study, this sensor can be compared with the performance of other sensors and tests, and the potential of sensor combinations for improved DPN assessment can be evaluated. If this measurement is found to be clinically useful, the system can be further developed with miniaturized impedance spectroscopy electronics integrated in the sock, with a semiautomated analysis and wireless transfer of test results. Several promising point-of-care devices for screening of diabetic peripheral neuropathy are available on the market, as reviewed in Selvarajah et al 2019. If comparable accuracy can be obtained with only bioimpedance spectroscopy, this method can potentially allow for a rapid test by a reusable small device with cheap manufacturing costs requiring no consumable parts. Another possibility may be if the method can supplement other tests by providing information more specific to the state of the skin.

## Conclusions

We present a prototype solution for impedance spectroscopy in diabetic foot assessment and early results comparing people with DPN and healthy controls. These early results indicate a potential to detect relevant skin changes associated with DPN, encouraging further studies with the big toe sensor in particular.
